# Effects of a dietary supplement on inflammatory marker expression in middle-aged and elderly hypertensive patients

**DOI:** 10.6061/clinics/2019/e890

**Published:** 2019-04-02

**Authors:** Jia Wang, Zhongxin Hong, Nan Wang, Li Wu, Bingjie Ding, Zhiwen Ge, Yanxia Bi, Wei Li

**Affiliations:** Department of Nutrition, Beijing Friendship Hospital, Capital Medical University, Beijing 100050, China

**Keywords:** Hypertension, Diet Composition, Inflammatory Markers, NF-κB, sICAM-1

## Abstract

**OBJECTIVES:**

We aimed to explore the effects of diet on the inflammatory response in middle-aged and elderly people with hypertension.

**METHODS:**

Thirty overweight or obese patients with stage one hypertension (age range, 45-75 years) were allocated to either the intervention or control group (n=15 per group; age- and sex-matched). Patients in the intervention group consumed a food powder supplement (100 g) instead of a regular meal. The control group maintained their normal dietary habits. This study lasted for six weeks. Blood pressure, inflammatory marker levels, and energy intake were measured before and after the study.

**RESULTS:**

After 6 weeks, the diet composition of the intervention group changed significantly (*p*<0.05). The intake of proteins, dietary fibre, monounsaturated fat, and polyunsaturated fat increased significantly (*p*<0.05), while the total energy intake trended towards an increase (*p*>0.05). In the control group, the total energy intake decreased significantly (*p*<0.05). The levels of nuclear factor-κB (NF-κB), soluble intercellular adhesion molecule-1 (sICAM-1) and high sensitivity C-reactive protein (hs-CRP) decreased, and adiponectin increased significantly in the intervention group (*p*<0.05); however, no significant changes were observed in the inflammatory marker levels of the control group. In the intervention group, systolic blood pressure decreased significantly (*p*<0.05), and diastolic blood pressure also exhibited a decreasing trend. No significant change in blood pressure was observed in the control group.

**CONCLUSION:**

The consumption of a food powder supplement can improve diet composition, decrease blood pressure and reduce inflammation in middle-aged and elderly overweight or obese hypertensive patients. The food powder supplement may also have an anti-atherosclerotic effect in hypertensive patients.

## INTRODUCTION

In recent years, an estimated 31.1% of the global population, or approximately 13.4-14.4 billion people, have been affected by hypertension 1. In our previous study, the prevalence of hypertension in the Zhangfang Village of the Fangshan District was found to be 59.1% 2. Studies have shown that the prevalence of hypertension in middle- and low-income countries (31.5%) is higher than that in high-income countries (28.5%) 1. Moreover, the prevalence of hypertension in rural areas (35.1%) is higher than that in urban areas (32.1%) 3. The rate of controlled hypertension in Canada and the United States is above 60% 4,5. The control of hypertension in China is significantly poorer than that in other countries.

The main treatment methods for hypertension are drug, lifestyle and dietary interventions. Several studies have evaluated drug treatment and the influence of lifestyle changes on hypertension; however, few studies have evaluated the influence of diet composition on hypertension. In fact, a balanced diet, nutritional supplements and a healthy lifestyle have equivalent blood pressure-lowering effects 6.

A wide body of evidence shows that hypertension is, in essence, a low-level inflammatory response 7. The inflammatory response plays an important role in the pathogenesis of hypertension 7. The activation of nuclear factor-κB (NF-κB) has been shown to contribute to vascular endothelial dysfunction and increased blood pressure levels 8. High sensitivity C-reactive protein (hs-CRP) is believed to be involved in the pathogenesis of insulin resistance, diabetes mellitus and metabolic syndrome, which includes hypertension 9; it has been suggested that elevated hs-CRP should be added to the criteria for the identification of metabolic syndrome. Serum intercellular cell adhesion molecule-1 (sICAM-1) is associated with atherosclerosis and influences blood pressure levels 10. Hence, suppression of the inflammatory response is a key challenge in the treatment of hypertension.

Hypertensive patients can benefit from a balanced diet 6. In contrast, an unbalanced diet can promote the inflammatory response and/or lack the components that prevent this inflammatory response, which can further lead to hypertension.

An unbalanced diet may contain high sodium, low potassium, high energy (e.g., high fat, high carbohydrate) and low protein. A high-sodium diet is a known risk factor for hypertension 11,12. A low-potassium diet elevates blood pressure 13. High energy intake is a risk factor for hypertension. When the energy intake exceeds the recommended intake by >20%, the risk of hypertension has been shown to increase 1.7-fold 14. Moreover, dietary sources of energy (proportion of energy from fats, proteins or carbohydrates) are also key dietary factors that may lead to elevated blood pressure. Fat is a pro-inflammatory nutrient, and high fat intake is a risk factor for hypertension 15. High carbohydrate intake is also associated with a high prevalence of hypertension, as shown in our preliminary study 16. Studies conducted overseas have also shown that compared to a low-fat diet, a low-carbohydrate diet can decrease the level of inflammatory markers 17, and the level of inflammatory markers is closely linked to hypertension 18. Inadequate protein intake can also cause high blood pressure. Sugar intake is also positively associated with hypertension 19.

Diets in rural areas are typically high in energy (high-carbohydrate diets composed of white rice and pickles), low in protein and devoid of fruits and vegetables 16. Diets in rural areas have higher sodium contents and lack protein, taurine, calcium, magnesium, phosphorus and other nutrients. This unbalanced diet is related to blood pressure 20.

Western guidelines for the prevention and treatment of hypertension include dietary recommendations such as the Dietary Approach to Stop Hypertension (DASH) diet (class B) 21,22 and the Japanese guidelines for hypertension management, which recommend 23 the Mediterranean diet. The Mediterranean dietary pattern includes increased intake of vegetables, fruits, fish and oils and reduced intake of cholesterol and saturated fatty acids. Alternatively, the hypertension guidelines in China recommend only restriction of the dietary intake of sodium and contain no other dietary recommendations 24. Therefore, we should pay full attention to dietary management as an integral component of the management of hypertension. Although the DASH diet and Mediterranean diet have a definite effect on reducing blood pressure, these dietary patterns are difficult to adopt in China, especially in rural areas.

The present study aimed to investigate the effects of a dietary supplement on blood pressure and inflammatory marker expression in middle-aged and elderly hypertensive people. We hope to develop a feasible dietary intervention model that can help reduce the prevalence of hypertension in rural areas of China.

## MATERIALS AND METHODS

### Participants

Thirty hypertensive patients who were overweight or obese (age range, 45 to 75 years) were enrolled in the study from 27 June 2015 to 15 August 2015. All of the patients were from the Zhangfang Village of the Fangshan District in Beijing.

The inclusion criteria were as follows: (1) body mass index (BMI) >24.0 kg/m^2^ (24.0<BMI<28.0 kg/m^2^ defined as overweight; BMI ≥28.0 kg/m^2^ defined as obese); (2) systolic blood pressure between 140 and 159 mmHg (1 mmHg=0.133 kPa), and/or diastolic blood pressure between 90 and 99 mmHg; and (3) diagnosis of stage 1 hypertension and absence of medical treatment for hypertension.

The exclusion criteria were as follows: (1) hepatic and renal insufficiency, (2) cognitive impairment, (3) mental abnormality, (4) pregnancy or lactation, (5) alcoholism (daily intake of liquor >150 g/d or beer >500 mL/d), (6) irregular dietary habits or inability of maintaining a dietary intervention. The patients were divided into the intervention group (IG) and control group (CG) and were matched by age, sex and BMI.

This trial was registered with the Chinese Clinical Trial Registry (Number ChiCTR1800014475). The study was approved by the ethical committee of the local hospital. Written informed consent was obtained from all participants.

### Materials

The dietary supplement used in this study is called “Special Food Powder from Nuts and Seeds (SFPNS)”. It was developed by the Department of Nutrition at the Beijing Friendship Hospital of Capital Medical University. The formulation is patented under national patent protection (patent number: 201310706278.6). The components of the dietary supplement include rye, black rice, walnuts, lotus seeds, black beans, yams, American ginseng and Chinese dates. The recommended daily intake of the dietary supplement is 77 g, which contains 14 g rye, 7 g black rice, 14 g walnuts, 14 g lotus seeds, 14 g black beans, 7.5 g yams, 2.5 g American ginseng, 4 g Chinese dates; and provides 293 kcal energy, 13 g protein (17.75%), 40 g carbohydrates (54.61%), and 9 g fat (27.644%).

### Study design

On 27 June 2015, all 30 participants were counselled about the dietary supplement and informed about the study methods before the beginning of this trial. All participants underwent an initial taste test of the dietary supplement. The period from 28 June 2015 to 4 July 2015 was the adaptation period. In this period, all participants in the two groups consumed their original diets, and the dietary intake over the course of three days (including 2 work days and 1 weekend day) and physical activity over the course of two days were recorded. On 4 July 2015, the blood pressure of the participants was measured, and the blood levels of inflammatory markers (including NF-κB, sICAM-1, hs-CRP and adiponectin) were determined; in addition, a detailed dietary survey was conducted 18. The intervention trial period was from 5 July 2015 to 15 August 2015. Participants in the IG were administered 77 g SFPNS every day instead of 100 g of their regular meal, while participants in the CG maintained their original diet. None of the participants in the two groups received any drug treatments; all participants maintained their original lifestyle during the study period. Six weeks later, blood pressure and the levels of inflammatory markers were measured again. Additionally, another detailed dietary survey was conducted.

### Biochemical assessment

After 12h of overnight fasting, 10 mL venous blood samples were obtained and immediately centrifuged at 2500 ×g for 10 min at 4°C. NF-κB, sICAM-1 and adiponectin were estimated via ELISA, and hs-CRP was measured by immune turbidimetry. These indices were measured by the Beijing Institute of Clinical Laboratory Limited.

### Diet survey

We used a 24-h dietary recall survey method to collect the participants' dietary histories. Subsequently, the integrated traditional Chinese and Western medicine nutritional treatment expert system (NCCW2011) nutrition analysis software was used to analyze the intake of total energy, carbohydrates, proteins, fat, dietary fibre, selenium, zinc, vitamin B_1_, monounsaturated fatty acids and polyunsaturated fatty acids.

### Statistical analysis

Prior to statistical analysis, the normality of the distribution of all variables was assessed using the Kolmogorov-Smirnov test, histograms and p-p plots. If needed, the data were log (ln) transformed to achieve a normal distribution. Descriptive statistics (mean, SD, and range) of the general characteristics of the study participants are reported. Between-group differences with respect to sex, smoking, and alcohol consumption were assessed by the chi-squared test. Data pertaining to dietary intake, lipid levels, age, physical activity and sleep duration were compared by *t* tests.

### Quality control method

During the study period, nutritionists conducted detailed dietary surveys and provided guidance to all participants. The nutritionists performed a telephone survey 2-3 times per week and detected dietary changes on time and guided the participants in accordance with the requirements of the study diet procedure. The village committee appointed a specific person to distribute the dietary supplement and established the registration procedure.

## RESULTS

All 15 participants in the IG tolerated the dietary supplement and experienced no allergic reactions. In total, 22 patients (10 patients in the IG, 12 patients in the CG) completed the study. In the IG, 5 participants withdrew from the study because of surgery, overseas travel, or personal reasons. In the CG, 3 participants withdrew because of personal reasons.

No significant between-group differences were observed with respect to sex, age, smoking, alcohol consumption, physical activity of sleep duration at baseline (*p*>0.05 for all; Table 1).

**Figure 1:**
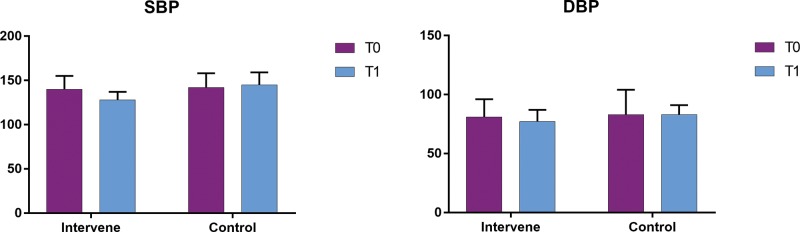
Comparison of blood pressure between the two groups. SBP, systolic blood pressure; DBP, diastolic blood pressure; Intervene, intervention group; Control, control group; T0, the data before the experiment; T1, the data after the experiment.

Before the experiment, the blood pressure levels in the two groups were not significantly different (*p*>0.05; Table 2 and Figure 1). After the 6-week experiment, the systolic blood pressure of the IG showed a significant decrease compared to that of the CG (*p*<0.05; Table 2 and Figure 1). The diastolic blood pressure also showed a decreasing trend; however, the between-group difference was not statistically significant (*p*<0.1; Table 2 and Figure 1). In the CG, the systolic and diastolic blood pressure at the completion of the experiment were not significantly different from the baseline levels (*p*>0.05; Table 2 and Figure 1).

**Figure 2:**
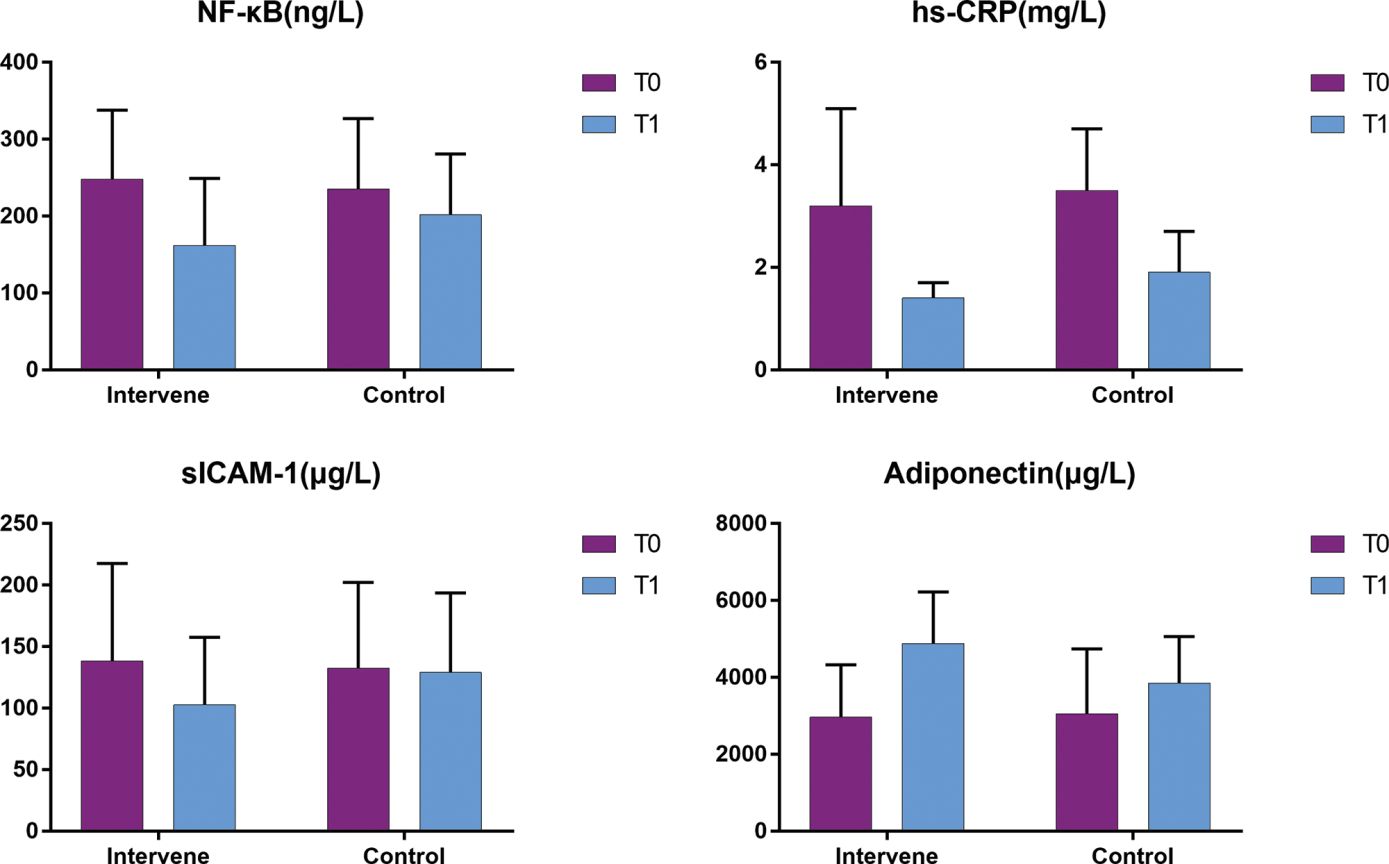
Comparison of inflammatory markers between the two groups. Intervene, intervention group; Control, control group; T0, the data before the experiment; T1, the data after the experiment.

The levels of inflammatory markers, including NF-κB, sICAM-1, hs-CRP and adiponectin, in the two groups before and after the experiment are summarized in Table 3 and Figure 2. Before the experiment, no significant between-group differences were observed with respect to the levels of inflammatory markers (all *p*>0.05). After the 6-week experiment, the levels of NF-κB, sICAM-1, and hs-CRP in the IG decreased significantly (all *p*<0.05), while the adiponectin level was significantly higher than the baseline level (*p*<0.05). In the CG, the levels of NF-κB and sICAM-1 showed a decreasing trend (*p*>0.05), while the adiponectin level showed an increasing trend (*p*>0.05); however, the changes were not statistically significant. In the CG, only the hs-CRP level showed a significant decrease (*p*<0.05).

**Figure 3:**
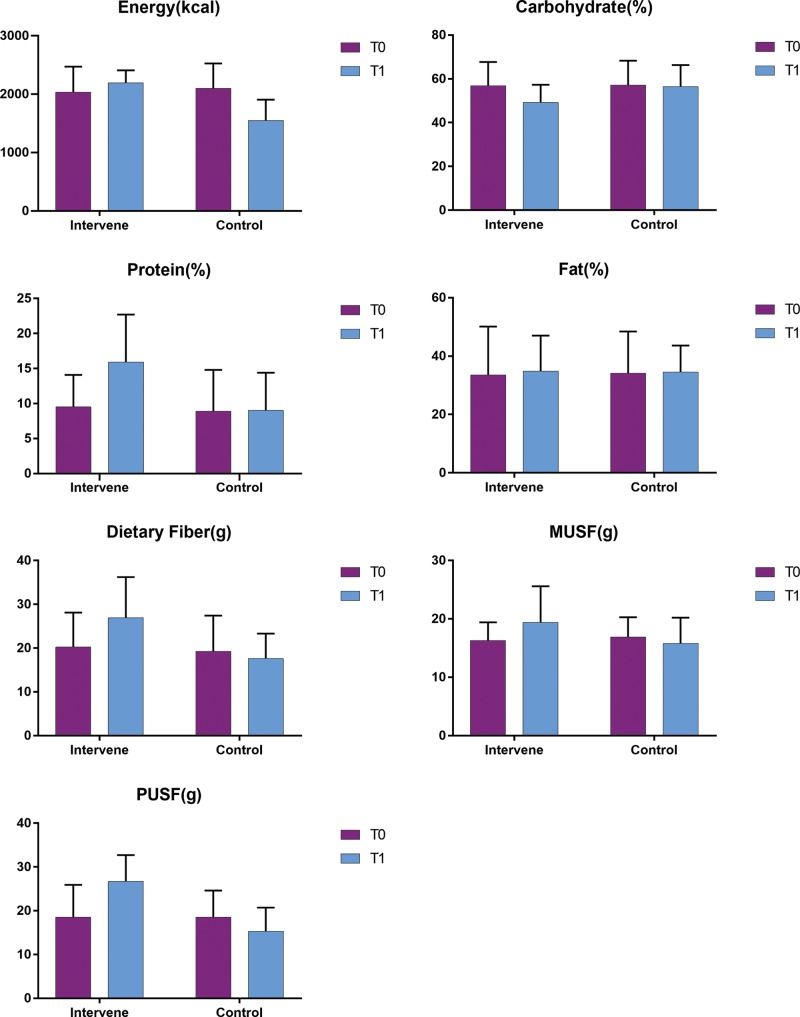
Comparison of total energy intake and proportions of different nutrients between the two groups before and after the study. Intervene, intervention group; Control, control group; T0, the data before the experiment; T1, the data after the experiment.

The comparisons of energy intake and the proportions of nutrient components between the two groups before and after the study are summarized in Table 4 and Figure 3. Before the experiment, the total energy and nutrient intakes showed no significant between-group differences (both *p*>0.05). Following the 6-week experiment, in the IG, the proportion of protein increased significantly (*p*<0.05), while the proportion of carbohydrates showed a decreasing trend (*p*>0.05). In addition, the intakes of dietary fibre, monounsaturated fatty acids, and polyunsaturated fatty acids increased significantly (*p*<0.05). In the CG, the energy intake decreased significantly (*p*<0.05); however, no significant change in the proportion of fibres, monounsaturated fatty acids, and polyunsaturated fatty acids was observed (*p*>0.05).

## DISCUSSION

In this study, we first demonstrated that dietary improvements (such as an increase in the proportion of proteins, increased intake of fibres and unsaturated fatty acids) without a reduction in total energy intake can reduce the levels of inflammatory markers in middle-aged and elderly hypertensive patients. In the study population, the levels of NF-κB, sICAM-1 and hs-CRP were decreased significantly, and the blood level of adiponectin increased significantly after consuming the dietary supplement produced by the Department of Nutrition at Beijing Friendship Hospital of Capital Medical University. In addition, the systolic blood pressure markedly declined, and the diastolic blood pressure slightly declined after the dietary supplement intervention. In addition, the reduction in total energy intake alone, without a concomitant change in diet composition, significantly reduced hs-CRP levels; however, no significant changes in systolic and diastolic blood pressure, NF-κB or sICAM-1 were observed.

Our results differ from those of previous studies. In a study by Witkowska 10 conducted on 22 obese postmenopausal women, the restriction of energy intake (1,200 kcal/d) and the consumption of a low-fat, high-carbohydrate diet led to a mean weight reduction of 6 kg, along with decreased hs-CRP and increased adiponectin levels. These results showed that in obese patients, restricted energy intake and a low-fat, high-carbohydrate diet can decrease the level of inflammatory markers and lead to weight loss. Some scholars have confirmed that improving the diet can decrease the level of inflammatory markers. Forsythe et al. 17 found that in metabolic syndrome patients, compared to an energy-restricted, low-fat, high-carbohydrate diet (LED), an energy-restricted, very low-carbohydrate diet (VLCKD) increased the level of arachidonic acid, improved inflammatory marker levels and significantly decreased the level of sICAM-1. In a study by Zou et al. 25, limiting the energy intake alone attenuated oxidative stress and decreased the production of soluble adhesion molecules. Medina-Remon et al. 26 reported that dietary supplementation with nuts can increase the intake of polyphenols and decrease the level of inflammatory markers (VCAM-1, ICAM-1, IL-6, TNF-α) and blood pressure. These findings are consistent with those of the present study. These results suggest that dietary improvements are more important than mere restriction of energy intake in middle-aged and elderly hypertensive patients.

The dietary supplement used in this study is rich in grains and beans; moreover, it has a higher content of protein, dietary fibre and unsaturated fatty acids than a meal of white rice and pickles. with the consumption of this dietary supplement effectively improved the diet composition in the IG. While there was no significant change in the total energy intake, the proportions of protein, dietary fibre, and unsaturated fatty acids increased significantly. In the CG, there was no dietary intervention; however, the total energy intake decreased. We believe that this is largely attributable to seasonal factors. The study period spanned from July to August, when the temperature and humidity in Beijing are very high. These factors likely affected the appetite of the participants and reduced their total energy intake; however, the dietary composition did not change in terms of the proportion of protein, dietary fibre, and unsaturated fatty acids. In contrast, the energy intake in the IG showed an increasing trend due to the consumption of the supplement, which is rich in nuts and seeds and can improve appetite. Therefore, in the IG, the participants had a higher energy intake 26.

Several likely mechanisms may explain the anti-inflammatory and antihypertensive effects of the dietary supplement. First, the consumption of the dietary supplement increased protein intake and concomitantly decreased in the proportion of carbohydrates consumed. A diet that is relatively low in carbohydrates can increase the level of ketones, which in turn may attenuate oxidative stress and improve vascular endothelial function. Second, the high fibre content in the dietary supplement can stabilize blood glucose levels and reduce cholesterol absorption in vivo. Third, unsaturated fatty acids can regulate blood lipid levels, which can effectively improve endothelial function, reduce the inflammatory reaction of blood vessels, and play a role in decreasing blood pressure 17. Therefore, in the CG, a reduction in the total energy intake without improvement in diet composition did not adequately reduce the inflammatory reaction. Moreover, sICAM-1 is a marker of vascular inflammation. The level of sICAM-1 can reflect the activation level of endothelial cells and the degree of atherosclerosis 25. Malnutrition is closely related to sICAM-1.

NF-κB and some protein molecules regulated by NF-κB, such as adhesion molecules, integrin, and E-selectin, participate in vascular inflammation and cause vascular endothelial dysfunction. Malnutrition, such as excessive consumption of saturated fatty acids, trans-fatty acids, and energy, can activate NF-κB and increase the level of adhesion molecules, whereas other molecules, such as α-linolenic acids, unsaturated fatty acids, selenium, and soluble dietary fibre, can inhibit NF-κB. Under normal conditions, in vascular endothelial cells, NF-κB is activated first, then levels of the adhesion molecules increase, activating NF-κB in mononuclear macrophages, which further increases the levels of adhesion molecules 27.

Adiponectin, which is an insulin-sensitizing hormone, can increase fatty acid oxidation and glucose uptake in skeletal muscle cells. Additionally, adiponectin can significantly increase the inhibitory effect of insulin on gluconeogenesis and inhibit glucose production in the liver; moreover, adiponectin is an important regulatory factor in lipid metabolism and glucose homeostasis. Plasma levels of adiponectin showed a negative correlation with the levels of triglycerides (TGs) and low-density lipoprotein cholesterol (LDL-C) and a positive correlation with the level of high-density lipoprotein cholesterol (HDL-C). Studies conducted by Ouch et al. 28 and Shibata et al. 29 have shown that adiponectin can decrease adhesion in monocytes and decrease the expression of many adhesion molecules in vascular endothelial cells, such as VCAM-1, ICAM-1 and E-selectin. NF-κB is an important factor that stimulates the transcriptional regulation of VCAM-1, ICAM-1 and E-selectin. Adiponectin may regulate the function of endothelial cells by inhibiting the NF-κB signalling pathway, and TNF-α may specifically inhibit this activation.

### The limitations of the study

The sample size was small, and the test period was six weeks, which is relatively short. The study was not completed in one season, and the season may have affected the appetite of the participants and, thus, the results. In our study, although the participants in the IG consumed the dietary supplement, their diets did not reach the standard of a high-protein, low-carbohydrate diet, which may have compromised the accuracy of the results. Further study is needed to validate these findings.

## CONCLUSION

In our study, the energy intake in the IG did not change; however, the diet composition improved. In the CG, the energy intake decreased significantly; however, the diet composition did not improve. Our experiment was carried out in the summer, and the weather had an important impact on the diet. Additional studies involving a reduced energy diet with simultaneous dietary composition improvements are required. Further studies will be conducted in which the participants will be provide with all of their food to control the total energy intake and improve the diet composition through the consumption of a dietary supplement, and then the changes in inflammatory marker levels and blood pressure will be observed.

In summary, in overweight and obese urban residents with hypertension, improving diet composition by consuming a special dietary supplement can reduce the expression of inflammatory markers and decrease blood pressure. The consumption of a dietary supplement may be recommended as a strategy for the control of hypertension.

## AUTHOR CONTRIBUTIONS

Hong Z contributed to the conception of the study. Wang N, Wu L, Ding B, and Ge Z contributed significantly to the analysis and manuscript preparation. Wang J performed the data analyses and wrote the manuscript. Bi Y and Li W helped guiding the analysis with constructive discussions. All authors approved the final version of the manuscript for publication.

## Figures and Tables

**Table 1:** General characteristics of the patients in the two groups.

Group	N	Sex (Male/Female)	Age (y)	Smoking@ (Y/N)	Alcohol^&^ (Y/N)	Exercise^#^ (h/d)	Sleep (h/d)
IG	10	5/5	57.7±8.2	5/5	5/5	0.9±0.3	7.4±2.1
CG	12	6/6	60.8±7.8	6/6	6/6	1.2±0.4	7.1±1.4
*T*		-*	-0.420	-*	-*	-0.519	0.861
*p*		1.000	0.701	1.000	1.000	0.560	0.401

Note: IG, intervention group; CG, control group; *Fisher's exact probability method; ^&^daily consumption of alcohol in any form; ^#^time spent exercising, including heavy physical labour and physical exercise.

**Table 2:** Comparison of blood pressure between the two groups.

		SBP				DBP			
Group	N	before	after	*t*	*p*	before	after	*t*	*p*
IG	10	140±15	128±9	2.998	**0.008**	81±15	77±10	1.989	0.069
CG	12	142±16	145±14	-0.812	0.425	83±21	83±8	0.269	0.790
*T*	-	-0.504	-3.014	-	-	-0.696	-1.859	-	-
*p*	-	0.602	0.007	-	-	0.492	0.089	-	-

Data are presented as the mean ± standard deviation (mmHg).

Note: SBP, systolic blood pressure; DBP, diastolic blood pressure; before, the data before the experiment; after, the data after the 6-week experiment; IG, intervention group; CG, control group.

**Table 3:** Comparison of inflammatory markers between the two groups.

		NF-κB (ng/L)				sICAM-1 (µg/L)			
Group	N	T0	T1	*t*	*p*	T0	T1	*t*	*p*
IG	10	248.2±89.7	161.7±87.3	2.218	**0.041**	138.5±79.1	102.7±54.8	2.201	**0.045**
CG	12	235.4±91.4	201.5±79.4	0.861	0.239	132.6±69.5	128.8±64.9	0.997	0.327
*t*	-	0.284	-1.521	-	-	0.197	-2.171	-	-
*p*	-	0.724	0.158	-	-	0.892	0.295	-	-

		Adiponectin (µg/L)				hs-CRP (mg/L)			
Group	N	T0	T1	*t*	*p*	T0	T1	*t*	*p*
IG	10	2964.1±1364.1	4874.3±1349.7	2.221	**0.039**	3.2±1.9	1.4±0.3	2.289	**0.036**
CG	12	3049.2±1695.1	3849.7±1210.4	1.391	0.189	3.5±1.2	1.9±0.8	2.105	**0.048**
*t*	-	-0.197	2.291	-	-	-0.695	-0.910	-	-
*p*	-	0.893	**0.035**	-	-	0.529	0.361	-	-

Data are presented as the mean ± standard deviation.

**Table 4:** Comparison of total energy intake and proportions of different nutrients between the two groups before and after the study.

Group	N	Energy (kcal)				Carbohydrates (%)			
		T0	T1	*t*	*p*	T0	T1	*t*	*p*
IG	10	2033.7±437.5	2196.5±211.5	-1.500	0.149	56.9±10.8	49.2±8.1	1.980	0.075
CG	12	2098.5±427.7	1549.2±357.3	3.138	**0.004**	57.1±11.2	56.5±9.8	0.658	0.514
*t*	-	-0.274	4.297	-	-	-0.326	-1.761	-	-
*p*	-	0.759	**<0.001**	-	-	0.787	0.092	-	-

Group	N	Protein (%)				Fat (%)			
		T0	T1	*t*	*p*	T0	T1	*t*	*p*
IG	10	9.5±4.6	15.9±6.8	3.407	**0.003**	33.5±16.6	34.9±12.1	-0.228	0.743
CG	12	8.9±5.9	9.0±5.4	-1.289	0.231	34.1±14.3	34.5±9.1	-0.591	0.528
*t*	-	0.295	5.874	-	-	-1.961	1.038	-	-
*p*	-	0.846	**<0.001**	-	-	0.076	0.310	-	-

Group	N	Dietary Fibre (g)				Monounsaturated Fatty Acids (g)			
		T0	T1	*t*	*p*	T0	T1	*t*	*p*
IG	10	20.2±7.9	26.9±9.3	-3.465	**0.003**	16.3±3.1	19.4±6.2	-2.140	**0.045**
CG	12	19.2±8.2	17.6±5.7	0.230	0.820	16.9±3.4	15.8±4.4	1.069	0.308
*t*	-	1.197	6.725	-	-	-0.552	2.085	-	-
*p*	-	0.322	**<0.001**	-	-	0.635	**0.049**	-	-

Group	N	Polyunsaturated Fatty Acids (g)							
		T0	T1	*t*	*p*				
IG	10	18.5±7.4	26.7±6.0	-3.434	**0.003**				
CG	12	18.5±6.1	15.3±5.4	1.429	0.166				
*t*	-	-0.056	4.906			-	-	-	-
*p*	-	0.956	**<0.001**			-	-	-	-

Note: IG, intervention group; CG, control group; T0, the data before the experiment; T1, the data after the experiment; carbohydrate (%), the proportion of total energy from carbohydrates; protein (%), the proportion of total energy from protein; fat (%), the proportion of total energy from fats.

## References

[1] Mills KT, Bundy JD, Kelly TN, Reed JE, Kearney PM, Reynolds K (2016). Global Disparities of Hypertension Prevalence and Control: A Systematic Analysis of Population-Based Studies From 90 Countries. Circulation.

[2] Wang J, Hong Z, Wu L, Gu Z, Bi Y, Ding B (2013). Risk factors of primary hypertension for middle aged and elderly people in Zhangfang Village of Fangshan District in Beijing. Chinese General Practice.

[3] Guo J, Yu C, Lyu J, Guo Y, Bian Z, Zhou H (2016). [Status of prevalence, awareness, treatment and control on hypertension among adults in 10 regions, China]. Zhonghua Liu Xing Bing Xue Za Zhi.

[4] Guo F, He D, Zhang W, Walton RG (2012). Trends in prevalence, awareness, management, and control of hypertension among United States adults, 1999 to 2010. J Am Coll Cardiol.

[5] McInnis NH, Fodor G, Moy Lum-Kwong M, Leenen FH (2008). Antihypertensive medication use and blood pressure control: a community-based cross-sectional survey (ON-BP). Am J Hypertens.

[6] Caligiuri SPB, Pierce GN (2017). A review of the relative efficacy of dietary, nutritional supplements, lifestyle, and drug therapies in the management of hypertension. Crit Rev Food Sci Nutr.

[7] Pietri P, Vlachopoulos C, Tousoulis D (2015). Inflammation and Arterial Hypertension: From Pathophysiological Links to Risk Prediction. Curr Med Chem.

[8] Pierce GL, Lesniewski LA, Lawson BR, Beske SD, Seals DR (2009). Nuclear factor-{kappa}B activation contributes to vascular endothelial dysfunction via oxidative stress in overweight/obese middle-aged and older humans. Circulation.

[9] Bagherniya M, Khayyatzadeh SS, Heidari Bakavoli AR, Ferns GA, Ebrahimi M, Safarian M (2018). Serum high-sensitive C-reactive protein is associated with dietary intakes in diabetic patients with and without hypertension: a cross-sectional study. Ann Clin Biochem.

[10] Witkowska AM (2005). Soluble ICAM-1: a marker of vascular inflammation and lifestyle. Cytokine.

[11] Fang NY (2016). High salt diet and salt sensitive hypertension. Chin J Geriatr.

[12] Pilic L, Pedlar CR, Mavrommatis Y (2016). Salt-sensitive hypertension: mechanisms and effects of dietary and other lifestyle factors. Nutr Rev.

[13] Dyer AR, Elliott P, Shipley M (1994). Urinary electrolyte excretion in 24 hours and blood pressure in the INTERSALT Study. II. Estimates of electrolyte-blood pressure associations corrected for regression dilution bias. The INTERSALT Cooperative Research Group. Am J Epidemiol.

[14] Tang ZZ, Chen XL, Han YB (2007). Research about relationship of dietary and nutrients intake with hypertension in residents of guangxi. Pract Prevent Med.

[15] Zhang JD, FU Q (2015). Meta analysis of China's rural population hypertension risk factors. Chin J Health Statistics.

[16] Wang J, Hong ZX, WU L (2016). Dietary pattern and pathogenesis of hypertension and middle aged and elderly people. Chinese General Practice.

[17] Forsythe CE, Phinney SD, Fernandez ML, Quann EE, Wood RJ, Bibus DM (2008). Comparison of low fat and low carbohydrate diets on circulating fatty acid composition and markers of inflammation. Lipids.

[18] Mitu F, Rezus E, Banu C, Jufã C, Mitu O, Dima-Cozma C (2014). Inflammatory markers in hypertensive patients and influence of some associated metabolic risk factor. Rev Med Chir Soc Med Nat Iasi.

[19] Brown IJ, Stamler J, Van Horn L, Robertson CE, Chan Q, Dyer AR (2011). Sugar-sweetened beverage, sugar intake of individuals, and their blood pressure: international study of macro/micronutrients and blood pressure. Hypertension.

[20] Hong ZX, Ding BJ, Wang J (2013). The role of food functional components on prevention and treatment of hypertension. Hebei Medical Journal.

[21] Daskalopoulou SS, Rabi DM, Zarnke KB, Dasgupta K, Nerenberg K, Cloutier L (2015). The 2015 Canadian Hypertension Education Program recommendations for blood pressure measurement, diagnosis, assessment of risk, prevention, and treatment of hypertension. Can J Cardiol.

[22] Mancia G, Fagard R, Narkiewicz K, Redon J, Zanchetti A, Bohm M (2013). 2013 ESH/ESC guidelines for the management of arterial hypertension: the Task Force for the Management of Arterial Hypertension of the European Society of Hypertension (ESH) and of the European Society of Cardiology (ESC). Eur Heart J.

[23] Kario K (2015). Key Points of the Japanese Society of Hypertension Guidelines for the Management of Hypertension in 2014. Pulse.

[24] Commission HMGatG-rLotPsRoCR (2015). Chinese high blood pressure at the grass-roots level management guidelines (revised in 2014)[J]. Chin J Hypertens.

[25] Zou Y, Jung KJ, Kim JW, Yu BP, Chung HY (2004). Alteration of soluble adhesion molecules during aging and their modulation by calorie restriction. FASEB J.

[26] Medina-Remón A, Casas R, Tressserra-Rimbau A, Ros E, Martínez-González MA, Fitó M (2017). Polyphenol intake from a Mediterranean diet decreases inflammatory biomarkers related to atherosclerosis: a substudy of the PREDIMED trial. Br J Clin Pharmacol.

[27] Pham TX, Lee JY (2016). Anti-Inflammatory Effect of Spirulina platensis in Macrophages Is Beneficial for Adipocyte Differentiation and Maturation by Inhibiting Nuclear Factor-kB Pathway in 3T3-L1 Adipocytes. J Med Food.

[28] Ouchi N, Walsh K (2012). Cardiovascular and metabolic regulation by the adiponectin/C1q/tumor necrosis factor-related protein family of proteins. Circulation.

[29] Shibata R, Ouchi N, Murohara T (2009). Adiponectin and cardiovascular disease. Circ J.

